# Diversity, structure, and synteny of the cutinase gene of *Colletotrichum* species

**DOI:** 10.1002/ece3.5998

**Published:** 2020-01-21

**Authors:** Ria T. Villafana, Sephra N. Rampersad

**Affiliations:** ^1^ Faculty of Science and Technology Department of Life Sciences Biochemistry Research Lab The University of the West Indies St. Augustine Trinidad and Tobago – West Indies

**Keywords:** *Colletotrichum*, cutinase diversity, evolution, pathogenesis, synteny

## Abstract

*Colletotrichum* species complexes are among the top 10 economically important fungal plant pathogens worldwide because they can infect climacteric and nonclimacteric fruit at the pre and/or postharvest stages. *C. truncatum* is the major pathogen responsible for anthracnose of green and red bell pepper fruit worldwide. *C. brevisporum* was recently reported to be a minor pathogen of red bell pepper fruit in Trinidad, but has recently been reported as pathogenic to other host species in other countries. The ability of these phytopathogens to produce and secrete cutinase is required for dismantling the cuticle of the host plant and, therefore, crucial to the necrotrophic phase of their infection strategy. In vitro bioassays using different lipid substrates confirmed the ability of *C. truncatum* and *C. brevisporum* isolates from green and red bell peppers to secrete cutinase. The diversity, structure and organization and synteny of the cutinase gene were determined among different *Colletotrichum* species. Cluster analysis indicated a low level of nucleotide variation among *C. truncatum* sequences. Nucleotide sequences of *C. brevisporum* were more related to *C. truncatum* cutinase nucleotide sequences than to *C. gloeosporioides*. Cluster patterns coincided with haplotype and there was evidence of significant positive selection with no recombination signatures. The structure of the cutinase gene included two exons with one intervening intron and, therefore, one splice variant. Although amino acid sequences were highly conserved among *C. truncatum* isolates, diversity “hot spots” were revealed when the 66‐amino acid coding region of 200 fungal species was compared. Twenty cutinase *orthologue*s were detected among different fungal species, whose common ancestor is Pezizomycotina and it is purported that these *orthologue*s arose through a single gene duplication event prior to speciation. The cutinase domain was retained both in structure and arrangement among 34 different *Colletotrichum* species. The order of aligned genomic blocks between species and the arrangement of flanking protein domains were also conserved and shared for those domains immediately located at the N‐ and C‐terminus of the cutinase domain. Among these were an RNA recognition motif, translation elongation factor, signal peptide, pentatricopeptide repeat, and Hsp70 family of chaperone proteins, all of which support the expression of the cutinase gene. The findings of this study are important to understanding the evolution of the cutinase gene in *C. truncatum* as a key component of the biotrophic–necrotrophic switch which may be useful in developing gene‐targeting strategies to decrease the pathogenic potential of *Colletotrichum* species.

## Graphical abstract

Low sequence diversity and high evolutionary conservation of the cutinase gene in Colletotrichum truncatum are key to this phytopathogen's infection strategy.

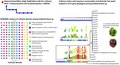

## 1 INTRODUCTION

Many plant pathogenic fungi have complex life cycles which enable them to interact differently with their hosts (Horbach, Navarro‐Quesada, Knogge, & Deising, 2011). As such, a two‐stage life cycle is typical of hemibiotrophic fungi: the initial biotrophic phase: the fungus grows and survives in a quiescent state to maintain host viability, therefore, the host remains asymptomatic and without host tissue destruction; the second necrotrophic phase: tissue is decomposed through cellular dismantling and destruction, assimilation of the contents of dead or dying cells which results in symptom manifestation and subsequent death of plant tissue (Brunner, Torriani, Croll, Stukenbrock, & McDonald, 2013; Stone, 2001).

*Colletotrichum* is one of the most economically important genera of plant pathogenic fungi with a membership of more than 200 species, most of which enjoy a broad host range worldwide, and cause anthracnose of fruit and vegetable crops in tropical and subtropical climates (Udayanga, Manamgoda, Liu, Chukeatirote, & Hyde, 2013). *Colletotrichum* species are hemibiotrophic plant pathogens that exhibit both forms of nutrient acquisition, that is, utilize sequential biotrophic and necrotrophic infection tactics to invade and cause disease in host plants (Alkan, Friedlander, Ment, Prusky, & Fluhr, 2015; Lee & Rose, 2010; O'Connell et al., 2012). Infection by fungal necrotrophs generally involves stages of conidial attachment, germination, host penetration, primary lesion formation, lesion expansion, and tissue maceration followed by sporulation (Prins et al., 2000). A comparative study involving the genome and transcriptome of *C. higginsianum* and *C. graminicola* indicated that (a) both fungal species possessed numerous pathogenicity‐related genes, with genetic footprints reminiscent of biotrophic and necrotrophic pathogens, (b) there are differences in the expression profile of these pathogenicity‐related genes, and (c) induction of gene expression is highly regulated and stage‐specific (Alkan et al., 2015; Damm, O'Connell, Groenewald, & Crous, 2014; Kleemann et al., 2012; Liu et al., 2013; O'Connell et al., 2012).

The interaction between a hemibiotroph and its plant host is highly specialized both structurally and physiologically such that the duration of the biotrophic or necrotrophic phase differs among hemibiotrophic pathogens and is dependent on physiological triggers of the host plant (Laluk & Mengiste, 2010). During fruit ripening, specific physiological changes occur in the host, including those involved in the natural ripening process, in recognition of the fungal pathogen, and in activation of the host's defense response (Alkan et al., 2015). Some of these physiological modifications include remodelling of the cell wall architecture (Brummell et al., 1999; Hückelhoven, 2007), accumulation of soluble sugar, altered bioactivity of phytoalexins, and phytoanticipins (Prusky, 1996); changes in the pH of the cytoplasm of the host plant cell (Prusky, Alkan, Mengiste, & Fluhr, 2013); and cuticle biosynthesis (Bargel & Neinhuis, 2005). Alterations of cuticle composition, structure, and deposition are involved in triggering the switch from a quiescent state to a necrotrophic state which in turn, allow fungal pathogens to penetrate the cuticle, infect and cause destruction of fruit tissue (Agudelo‐Romero et al., 2015; Alkan et al., 2015; Bhadauria et al., 2013; Blanco‐Ulate et al., 2014). These changes are regulated by a complex interplay of hormonal signals which affect both plant defense responses and resistance to pathogen invasion (Giovannoni, 2001; Seymour, Østergaard, Chapman, Knapp, & Martin, 2013; Voisin et al., 2009).

The cuticle is considered to be a “lipidized cell wall region” that is composed of several compounds, with cutin being the most abundant, followed by cuticular waxes which are a combination of organic solvent‐soluble, very long‐chain fatty acid compounds (Lara, Belge, & Goulao, 2015; Martin & Rose, 2014). As a physical barrier, the cuticle surrounds the epidermis of fruits and serves a number of functions including (a) to protect the plant against the physical, environmental and biological stresses and pathogen invasion, (b) to reduce the effects of internal water loss, and (c) to maintain plant organ integrity by providing mechanical support (Belge et al., 2014; Chen et al., 2013; Lara, Belge, & Goulao, 2014). In pathogenicity tests, *C. truncatum* isolates are pathogenic on *Capsicum* fruits after wounding the fruit surface, and most produced a low level of infection on nonwounded fruit (De Silva et al., 2019; Ramdial & Rampersad, 2015). The life cycle of *C. truncatum* in *Capsicum* sp. included biotrophic and necrotrophic phases of colonization with high susceptibility to lesion development during fruit ripening stages (Ranathunge, Mongkolporn, Ford, & Taylor, 2012; Ranathunge & Sandani, 2016). In climacteric fruits, changes in cuticle thickness and composition change during ripening (Martin & Rose, 2014). However, in nonclimacteric fruits, like bell pepper, once maximum cutin monomer levels are reached during development, it decreases steadily as the fruit ripens (Kosma et al., 2010). This demonstrates the significance of the cuticle to infection by *Colletotrichum* spp. (Auyong, Ford, & Taylor, 2015).

The cuticle also has active roles in host defense signalling in addition to local and systemic resistance against a variety of pathogenic fungi. Fungal spores adhere to the surface of the host plant which signals production of low levels of cutinase activity which produces small amounts of cutin monomers (Woloshuk & Kolattukudy, 1986). Fungal sensing of cutin monomers leads to (a) production of high levels of cutinase which is required for penetration in *Fusarium* sp. (Woloshuk & Kolattukudy, 1986), (b) induction of germination and appressorium formation in *Magnaporthe grisea* and *Erysiphe graminis* (Francis, Dewey, & Gurr, 1996; Gilbert, Johnson, & Dean, 1996), (c) activation of gene expression of lipid‐induced protein kinase (LIPK) which is essential for infection structure formation in *C. trifolii* (Dickman, Ha, Yang, Adams, & Huang, 2003). With respect to induction of typical pattern‐triggered immunity (PTI) responses and upregulation of defense‐related genes, recognition of pathogen‐associated molecular patterns (PAMPs) and damage‐associated molecular patterns (DAMPs), enhance resistance to both biotrophic and necrotrophic fungal aggressors (Choi & Klessig, 2016; Kauss, Fauth, Merten, & Jeblick, 1999; Mengiste, Alfen, Leach, & Lindow, 2012; Schweizer, Jeanguenat, Whitacre, Métraux, & Mösinge, 1996). *C. gloeosporioides* can also induce biosynthesis of ethylene, which is involved in the breaker stage of tomato fruit ripening, that is, a “break” in color from green to tannish‐yellow, pink, or red (Alkan et al., 2015; Blanco‐Ulate, Vincenti, Powell, & Cantu, 2013; Seymour et al., 2013). In this way, the fungus can manipulate the fruit ripening process to engage the switch from biotroph to necrotroph. Thus, the cuticle is a valuable research target for development of early sensing of fungal pathogens and activation of plant defense responses.

Cutinases (EC: 3.1.1.74) are members of the alpha/beta hydrolase family of lipases (Kolattukudy, 1984; Longhi & Cambillau, 1999). The enzyme is able to hydrolyze fatty acids esters and emulsified triacylglycerol as efficiently as lipases, and therefore, it is considered an intermediate between esterases and lipases (https://www.ebi.ac.uk/enzymeportal/ec/3.1.1.74) (Nyyssölä, 2015). The enzyme plays an active role in (a) carbon acquisition for saprophytic growth, (b) adhesion of fungal structures to the host surface, and (c) the early stages of fungal penetration (Auyong et al., 2015). Cutin monomers trigger expression and synthesis of cutinases required for fungal penetration into the plant tissues. Cutinases, therefore, have a critical role in plant surface signalling that elicits differentiation of those fungal structures required for infection (Belbahri, Calmin, Mauch, & Andersson, 2008).

Bell pepper (*Capsicum annuum* var. *grossum* (L.) Sendt.) is a member of the Solanaceae family and is one of the most widely cultivated vegetable crops in the world. Over the last decade, the world production and consumption of bell peppers have been steadily increasing. More than 70% of the world's bell peppers are produced in China (FAO, 2017, http://www.fao.org/faostat/en/#data/QC). In Trinidad, bell pepper is grown year‐round and is among the top 10 agricultural commodities in the country. Production, in terms of yield and cost, is directly affected by fruit rot caused primarily by *C. truncatum* (syn. *C. capsici* ‐ Damm, Woudenberg, Cannon, & Crous, 2009; Ramdial & Rampersad, 2015) and more recently by *C. brevisporum*, albeit with lower incidence compared to *C. truncatum* (Villafana, Ramdass, & Rampersad, 2019). *C. truncatum* was the predominant species of *Colletotrichum* associated with anthracnose of chili in Asia and is widely distributed throughout Asia, Australia, and South America (De Silva, Ades, Crous, & Taylor, 2017; De Silva et al., 2019; Diao et al., 2017; Mongkolporn & Taylor, 2018; Sharma, Kumar Pinnaka, & Shenoy, 2014). Multilocus phylogeny revealed that the recently characterized *Colletotrichum magnum* species complex consists of nine closely‐related species which includes *C. brevisporum* (Damm et al., 2019). *C. brevisporum* has also been reported as one of the causal agents of anthracnose disease in chili fruit in China and Brazil and in bell pepper fruit in Trinidad (De Silva, Crous, Ades, Hyde, & Taylor, 2017; Diao et al., 2017; Liu et al., 2016; Villafana et al., 2019).

It is hypothesized that (a) *C. truncatum* and *C. brevisporum* are hemibiotrophs that are capable of secreting cutinase as part of their infection strategy, (b) the single cutinase gene structure and arrangement identified in *C. truncatum* is highly conserved at both the level of the nucleotide and amino acid sequence as opposed to other fungal species with multiple copies of this gene with divergent functions, (c) the cutinase domain has no relationship to synteny of other protein domains flanking this cutinase domain as no information is currently available in the literature concerning flanking amino acid sequences. This study was, therefore, undertaken to (a) assess the ability of *C. truncatum* and *C. brevisporum* isolates from green and red bell peppers from Trinidad to produce and secrete cutinase based on in vitro bioassays, and (b) determine the diversity, structure and organization and synteny of cutinase gene sequences and amino acid sequences among different *Colletotrichum* species and within *C. truncatum* isolates as a major fungal pathogen of green and red bell pepper fruit worldwide. This work will increase our understanding of the evolution of cutinase genes in fungal–plant host interactions which can be used to develop strategies for disruption of cutinase gene target(s) to reduce diseases caused by these fungal pathogens.

## 2 MATERIALS AND METHODS

### 2.1 Collection of isolates

Fifty‐two infected green and red bell pepper fruit with anthracnose lesions were collected from fields across Trinidad. The location of the lesions on the surface of the fruit was recorded according to three regions: the shoulder (region surrounding the peduncle and calyx); the face (largest surface of the fruit); and bottom (apex either acute or truncated). Bell pepper fields located in the main growing areas in Trinidad were visited: Aranguez (north and south), Orange Grove, Maloney, Caura, Caroni, Bon aventure, Penal, Mayo. Thin, transverse slices of the pericarp ~1 cm in length were then cut at the shoulder and face of bell pepper fruit and mounted on slides. Sudan III was used to stain the cuticle an orange to red color for microscopic visualization (Yeung & Chan, 2015).

### 2.2 Isolation of fungal pathogens

The bell pepper fruits were surface sterilized by rinsing in 70% ethanol for 1 min followed by another rinse in 0.6% sodium hypochlorite solution for 1 min. Samples were then washed three times in sterilized distilled water and dried on sterilized tissue paper. 4‐mm^3^ blocks of fruit tissue were removed from the margins of the lesions and transferred to potato dextrose agar (PDA) media (Oxoid Ltd., Thermo Fisher Scientific, Inc., USA) supplemented with 50 mg/L streptomycin, tetracycline, and chloramphenicol. Plates were incubated for seven days in the dark at 25°C. Monoconidial cultures of *C. truncatum*, and *C. brevisporum* were subsequently obtained and maintained on PDA at 4°C for temporary storage, and as conidial suspensions in 50% glycerol at −70°C for long‐term storage. The identities of *C. truncatum* and *C. brevisporum* cultures were confirmed using comparisons of multiple gene sequences (internally transcribed spacer region, ITS1‐5.8S‐ITS2; beta tubulin, TUB2; Actin, ACT‐GenBank Accession Nos. MG822830‐2, KJ780718, KR029613, MG827234‐5, MG839690‐1, MG870320‐1) after PCR amplification of total genomic DNA extracted from each fungal culture according to published protocols (van Poucke et al., 2012; White, Bruns, Lee, & Taylor, 1990).

### 2.3 Cutinase activity

#### 2.3.1 Tween‐20 and tributyrin opacity test

Gel diffusion bioassays, for example, the Tween and tributyrin opacity test, are based on the ability of lipase‐secreting microbes to break down the lipid substrate incorporated into solid media (see review by Lanka & Latha, 2015). These tests minimize the cost of screening and protect the test microorganisms from the inhibitory effects of various indicator dyes and lipase activity is identified as a clear or turbid zone around the colonies after incubation (Lanka & Latha, 2015). The level of enzyme activity can be evaluated by measuring the diameter of the halo around the colonies. In this study, for the Tween‐20 test, a calcium salt‐free Bacto agar (Oxoid Ltd., Thermo Fisher Scientific Inc.) medium containing Tween‐20 (Sigma Aldrich Inc.) was used (Slifkin, 2000). A 4‐mm^3^ block taken from the advancing mycelial edge of an actively growing culture of each isolate (eight isolates each of *C. truncatum*, and *C. brevisporum*) was plated onto this medium in duplicate. The presence of a white crystalline precipitate around the colony indicated secreted lipase activity. The colony size was measured after three days. The test was repeated.

### 2.4 Statistical analyses

Data were analyzed using IBM SPSS Statistics version 20. Tables outlining summary statistics for colony diameter (mm) on Tween‐20 and tributyrin, respectively, as well as for halo size (mm) on tributyrin. Analysis of variance tests (One‐way ANOVA) was carried out for “Colony diameter (mm)* Species” for Tween‐20 and tributyrin samples, respectively, and “Halo size (mm)* Species” for tributyrin samples and the appropriate tables were extracted.

### 2.5 Rhodamine test

Use of a fluorescent indicator dye such as Rhodamine B or G in the presence of olive oil as a lipid substrate has been used to determine lipase‐positive microbes (Lanka & Latha, 2015). Hydrolysis of a lipid substrate (e.g., olive oil) by secreted lipase results in the production of free fatty acids which interacts with Rhodamine fluorescent dye in the medium and causes the formation of yellow to orange fluorescent colonies which are visible upon UV irradiation. A 4‐mm^3^ block taken from the advancing mycelial edge of an actively growing culture of each isolate (eight isolates each of *C. truncatum*, and *C. brevisporum*) was plated onto this medium in duplicate as previously described for the Tween‐20 and tributyrin opacity tests. This test was repeated. Observation of a fluorescent yellow to orange colonies under UV light was recorded after six days.

### 2.6 DNA extraction, PCR amplification, and sequencing

DNA was extracted from 21 actively growing colonies using the Maxwell^®^‐16 automated DNA extraction system (Promega) based on magnetic bead capture DNA extraction according to the manufacturer's instructions. PCR amplification was carried out using published protocols for culture identification with modifications (O'Donnell et al., 2004; White et al., 1990). The primers and their use in this study are described in Table 1. For a single 25 µl reaction using GoTaq® Green Master Hot Start Taq DNA Polymerase (Promega Corporation) the PCR components included 1 × master mix, 50 pmoles of each primer (Integrated DNA Technologies), and 5 µl of the 1:4 diluted DNA sample. PCR amplification thermal conditions consisted of an initial denaturation of 5 min at 94°C followed by 35 cycles of 1 min at 94°C, annealing temperature and duration according to primer sequences, 1 min at 72°C with a final extension of 5 min at 72°C. Amplicons were sequenced at MCLAB (Molecular Cloning Laboratories). Sequence identities were verified using the BLAST algorithm in NCBI. Representative cutinase sequences for *C. truncatum* and *C. brevisporum* were deposited in GenBank (GenBank Accession Nos. MN473062 and MN473063, respectively). Twenty‐one representative cccut‐F/R‐generated nucleotide sequences were used in the final data set.

**Table 1:** Primers used in this study

Fungus	Gene target	Primer name	Primer orientation	Primer sequence (5′−3′)	Expected band size (bp)	Reference
*C. gloeosporioides *sensu lato	Species complex‐specific ITS	CgInt	Forward	GGCCTCCCGCCTCCGGGCGG	450	Mills, Sreenivasaprasad, and Brown (1992)
		ITS4	Reverse	TCCTCCGCTTATTGATATGC		White et al. (1990)
Fungi	Fungi ITS	ITS5	Forward	GGAAGTAAAAGTCGTAACAAGG	550–650	White et al. (1990)
		ITS4	Reverse	TCCTCCGCTTATTGATATGC		White et al. (1990)
*C. truncatum*	Species‐specific ITS	Ccap‐F	Forward	GTAGGCGTCCCCTAAAAAGG	394	Torres‐Calzada et al. (2011)
		Ccap‐R	Reverse	CCCAATGCGAGACGAAATG		
*C. gloeosporioides *sensu lato	Cutinase	CgCUT‐F	Forward	ATCAGGGTCAGCTAGGTTAGT	308	This study
		CgCUT‐R	Reverse	GGATCGTGAGGCCCTATTTATG		
*C. truncatum*	Cutinase	CcCUT‐F	Forward	AGAGTTTCTTCCGACCATTCC	729	This study
		CcCUT‐R	Reverse	GCCCTGTTATAGGAGTCAGTTATC		
*C. gloeosporioides *sensu lato	partial Actin	ACT−512	Forward	ATGTGCAAGGCCGGTTTCGC	300	Carbone and Kohn (1999)
		ACT−738	Reverse	TACGAGTCCTTCTGGCCCAT		
Filamentous Ascomycetes	partial β‐Tubulin	Bt2a	Forward	GGTAACCAAATCGGTGCTGCTTTC	170	Glass and Donaldson (1995)
		Bt2b	Reverse	ACCCTCAGTGTAGTGACCCTTGGC		

Cutinase‐specific cccut‐F/R primers were designed against three *C. truncatum* sequences mined from GenBank (GenBank Accession Nos. M18033, HQ406775 and XM_007602402) using the IDT DNA oligo primer quest tool (https://www.idtdna.com/PrimerQuest/Home/Index). Amplicons were sequenced directly (MCLAB, CA, USA), and sequence identities were verified by BLAST analysis.

### 2.7 Cutinase nucleotide sequence diversity

Nucleotide sequences were analyzed for sequence diversity and evolutionary maintenance. Sequences were aligned using MAFFT (Multiple Alignment using Fast Fourier Transform; https://www.ebi.ac.uk/Tools/msa/mafft/; Katoh & Standley, 2013), edited in Bioedit (Hall, 1999), and the FASTA alignment files were analyzed in DnaSP (DNA Sequence Polymorphism DnaSP version 6.12.03; Librado & Rozas, 2009; Rozas, 2009) to evaluate DNA polymorphism and evidence of selection.

### 2.8 Cluster analysis

Cluster analysis of the aligned nucleotide sequences was carried out in MEGA (MEGA7: Molecular Evolutionary Genetics Analysis version 7.0; Kumar, Stecher, & Tamura, 2016) using the maximum likelihood algorithm under the GTR + G + I nucleotide substitution model (based on a test of nucleotide substitution model in MEGA7) with 1,000 replicates. Four reference sequences from GenBank were included in the alignment (GenBank Accession Nos. M18033 and HQ406775–*C. truncatum*; KP331429 and M21443–*C. gloeosporioides*; there were no reference cutinase sequences for *C. brevisporum*).

### 2.9 Cutinase amino acid sequence diversity

Based on the findings of the cluster analysis, nucleotide sequences of the cccut‐F/R data set were translated to single amino acid sequence and the correct reading frame was identified using the ExPaSY translate tool (https://web.expasy.org/translate/). Two data subsets were analyzed: data subset 1‐ cutinase amino acid sequence (derived from translation of amplified nucleotide sequences by cccut‐F/R primers) curated to 219 amino acids in length and which included exonic and intronic sequences; data subset 2‐cutinase amino acid sequence (derived from translation of amplified nucleotide sequences by cccut‐F/R primers) curated to 66 amino acids in length and only included exonic sequences. The alignment, query coverage, and percent identity were then examined for “within” *Colletotrichum* species (intraspecific diversity) and “among” *Colletotrichum* species (interspecific diversity).

Multiple nucleotide sequences were translated to amino acid sequences using the EMBOSS Transeq tool (https://www.ebi.ac.uk/Tools/st/emboss_transeq/). The multiple sequences were then aligned using EMBL‐EBI Clustal Omega (https://www.ebi.ac.uk/Tools/msa/clustalo/). Clustal Omega is a multiple sequence alignment program that uses seeded guide trees and HMM profile–profile techniques to generate alignments among three or more sequences. This alignment was then used to generate an amino acid conservation plot in BioEdit (Hall, 1999), and this conserved sequence was used in subsequent analyses. Protein domain superfamilies in CATH‐Gene3D (http://www.cathdb.info/search/; Dawson et al., 2016; Lewis et al., 2017) have been subclassified into functional families (or FunFams), which are groups of protein sequences and structures with a high probability of sharing the same function(s). Therefore, the functionally important residues in a family are also expected to be highly conserved. Information on conserved positions in CATH‐Gene3D cutinase amino acid sequence alignment was analyzed (http://www.cathdb.info/version/v4_2_0/superfamily/3.40.50.1820/funfam/115401/alignment?task_xml:id=d1db710a5a097f00badbddcb35a91fc5).

### 2.10 Lineage of cutinase protein based on sequence, structure, and functional diversity

CATH‐Gene3D provides information on the evolutionary relationships of protein domains through sequence, structure, and functional annotation data. The taxonomic lineage of cutinase protein was hypothesized using CATHDB version 4.2 (http://cathdb.info/version/v4_2_0/superfamily/3.40.50.1820/funfam/115401/taxonomy_sunburst) starting with Eukaryota and ending with *C. truncatum*.

### 2.11 Structure, organization, and synteny of cutinase gene among 34 *Colletotrichum* species and among different fungal species

The structure and organization of the cutinase gene and protein domain were analyzed for 34 available *Colletotrichum* genomes in the MycoCosm fungal genomes database and portal of the Joint Genome Institute (https://mycocosm.jgi.doe.gov/mycocosm/home; Grigoriev et al., 2011, 2014).

## 3 RESULTS

**Figure 1:**
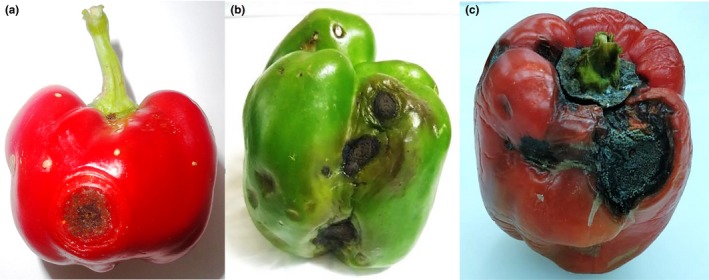
Symptoms of (a) *Colletotrichum brevisporum* infection in red bell pepper fruit; (b) *C. truncatum* infection in green bell pepper fruit; (c) *C. truncatum* infection in red bell pepper fruit

**Figure 2:**
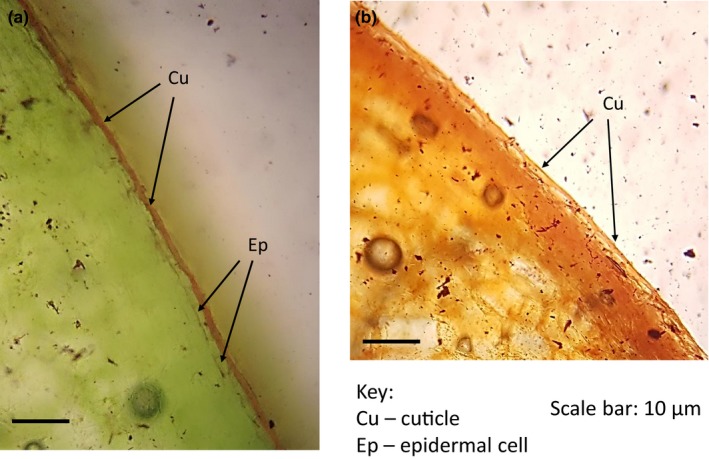
Histological section of bell pepper fruit showing the relative cuticle integrity and thickness in (a) green bell pepper fruit and (b) red bell pepper fruit

Lesions caused by *C. truncatum* were recorded at the shoulder, face, and bottom of the fruit (Figure 1). The frequency of lesion according to location on fruit surface was 44.2% on the shoulder, 50.0% on the face and 53.8% on the bottom. As such, the frequency distribution showed no preference for lesion development at a specific location on the fruit surface (Table A1). Figure 2 shows the difference in cuticle integrity and thickness in green versus red bell pepper fruit.

### 3.1 Cutinase bioassays

**Figure 3:**
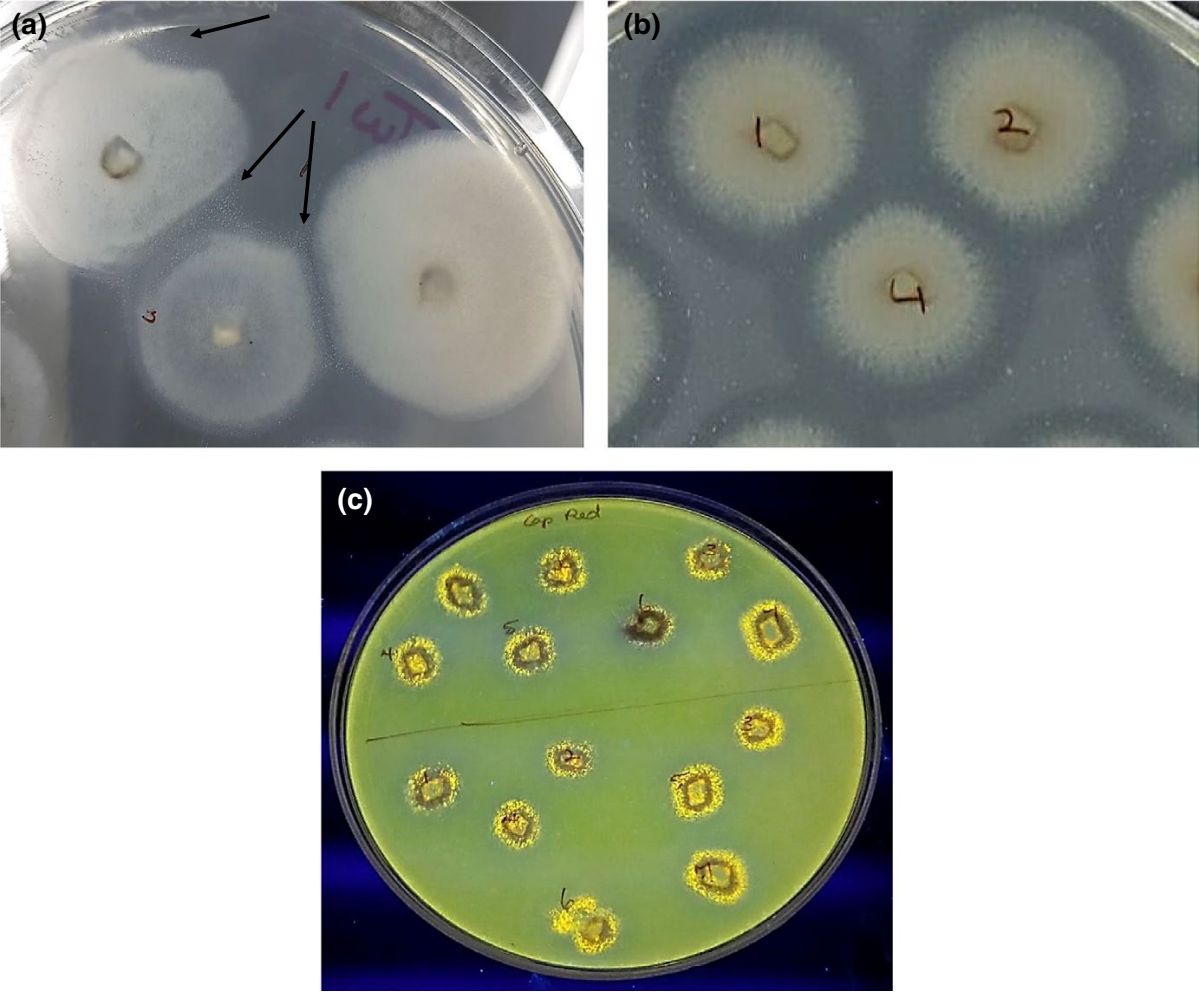
Lipid substrate bioassays; (a) arrows point to white crystalline precipitate on Tween‐20 medium; (b) zone of clearance (halo) around colonies producing secreted lipase (alpha/beta hydrolase/cutinase) on tributyrin medium; (c) yellow–orange fluorescent colonies on Rhodamine medium

Tween‐20 Opacity Test: A white crystalline precipitate was observed for all isolates of *C. truncatum* and *C. brevisporum* (Figure 3a). Table A2 outlines the summary of statistics for colony diameter (mm) for each *Colletotrichum* species on Tween‐20. *C. truncatum* had the larger colony diameter (mm) at 31.84 mm, as well as the higher mean value at 19.53 mm, while *C. brevisporum* had the smaller colony diameter at 9.21 mm on Tween‐20 media. Based on the analysis of variance (ANOVA) of colony diameter (mm) values for *C. truncatum and C. brevisporum* on Tween‐20 media, there was a significant difference in colony diameter (mm) at *p* = .007 (Table A3).

Tributyrin Test: The development of a zone of clearance (halo) for all isolates plated on tributyrin agar indicated lipase activity for *C. truncatum* and *C. brevisporum*. (Figure 3b). Summary of statistics for colony diameter (mm) for each *Colletotrichum* species on tributyrin media (Table A4) indicated that *C. truncatum* had the larger colony diameter (mm) at 15.56 mm, as well as the higher mean diameter value at 6.18 mm. The maximum and minimum halo sizes (mm) measured for *C. truncatum* were 12.74 mm and 3.42 mm, respectively. *C. truncatum* halo diameter also had higher mean value at 8.71 mm (Table A5). Analysis of variance of colony diameter for the two *Colletotrichum* species indicated that there was a significant difference in colony diameter (mm) between *C. truncatum* and *C. brevisporum*, (*p* = .021; Table A6). However, there was no significant difference in halo diameter (mm) for *C. truncatum and C. brevisporum* (Table A7).

Rhodamine Test: Yellow–orange‐colored fluorescent colonies were observed under UV light, which confirmed secreted cutinase activity for all isolates of *C. truncatum* and *C. brevisporum* (Figure 3c).

### 3.2 Cutinase nucleotide and amino acid sequence analyses

#### 3.2.1 Nucleotide sequence analysis

Nucleotide sequences generated from cccut‐F/R primers (trimmed to a final length of 656 bases) were identified as cutinase gene with 100% sequence coverage, 99.54% and 99.24% sequence identity with an E‐value of 0.0 to *C. truncatum* (GenBank Accession Nos. HQ406775 and M18011).

The degree of DNA polymorphism and related parameters are presented in Table 2. The nucleotide sequence generated by the cccut‐F/R primer pair consists of exonic and intronic sequences. There were no recombination signatures detected in the aligned nucleotide sequence data set. Tests of neutrality indicated that the nucleotide sequences were under significant (*p* ≤ .02) positive selection based on Fu and Li's D* statistic (Fu & Li, 1993).

**Table 2:** DNA polymorphism analysis of aligned nucleotide sequences generated from cccut‐F/R primers that target the cutinase gene in *Colletotrichum truncatum* isolates

	cccut‐F/R data set
*N*	21
DNA polymorphism	
No. of polymorphic sites, *S*	17
No. of mutations, *Eta*	17
Average no. of nucleotide differences, *k*	4.886
Nucleotide diversity, *Pi*	0.00748
Haplotype analysis	
No. of haplotypes, *h*	3
Haplotype diversity, H*d*	0.643
Tests of neutrality	
Tajima's *D* statistic	0.12559
Fu and Li's D* statistic	1.55120*
Fu and Li's F* statistic	1.3145
Recombination events, *Rm*	0

* Significant at *p* ≤ .02

#### 3.2.2 Cluster analysis

**Figure 4:**
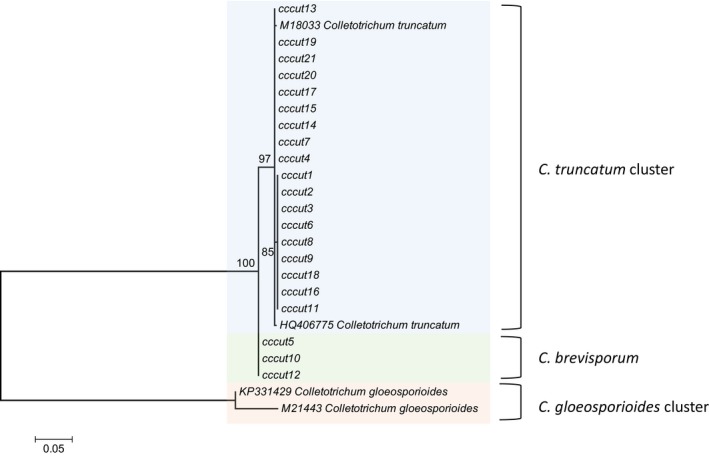
Cluster analysis of cutinase nucleotide sequences designed in this study using the maximum likelihood method based on the GTR + G + I model. The tree with the highest log likelihood is shown. The rate variation model allowed for some sites to be evolutionarily invariable. The tree is drawn to scale, with branch lengths measured in the number of substitutions per site

For the cccut‐F/R‐generated nucleotide sequence analysis, the clusters were strongly supported (minimum bs ≥ 85%) and were constructed according to haplotype: two haplotypes for the *C. truncatum* sequences, one haplotype for the *C. brevisporum* sequences, and two haplotypes that represented the two *C. gloeosporioides* sequences (Figure 4). The structure of each haplotype cluster was largely polytomic indicating no sequence diversity within a particular cluster. Clustering was irrespective of the maturity of the bell peppers fruit, that is, whether red or green.

#### 3.2.3 Cutinase amino acid sequence diversity

**Figure 5:**
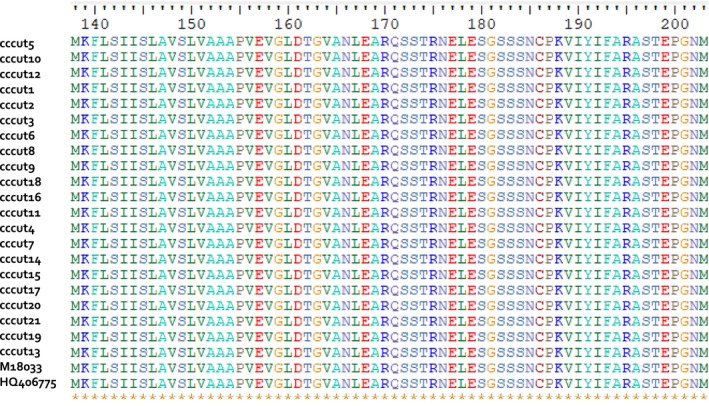
Conservation plot of cutinase amino acid sequence belonging to *Colletotrichum truncatum* isolates from Trinidad and *C. truncatum* reference sequences from GenBank

Cutinase amino acid sequence was invariable at the intraspecific level with 100% amino acid sequence identity at 100% coverage of the query sequence with *C. truncatum* (UniProtKB ‐ P10951; GenBank Accession No. ADQ27862). However, variability existed at the interspecific level with 83.3% sequence identity to *C. gloeosporioides* (GenBank Accession Nos. AAL38030 and AKH80819) at 100% of the query sequence. “Among species” amino acid sequence variability was at minimum for *C. gloeosporioides* at 83.3% and maximum for *C. spinosum* at 74.2% (Table 3). For all *C. truncatum* and *C. brevisporum* amino acid sequences, there was 100% conservation of the core coding region that corresponded to the cutinase domain despite nucleotide sequence variation in the same region (Figure 5).

**Table 3:** Diversity of the cutinase amino acid sequence among 17 *Colletotrichum* species

*Colletotrichum* species	GenBank Accession	Sequence identity %	Aligned aa sequence length	bit score
*C. truncatum*	P10951.1	100	66	2.42E−37
*C. capsici*	ADQ27862.1	100	66	3.04E−37
*C. gloeosporioides*	AAL38030.1	83.333	66	1.88E−26
*C. gloeosporioides*	AKH80819.1	81.818	66	4.49E−26
*C. gloeosporioides*	P11373.1	81.818	66	5.06E−26
*C. simmondsii*	KXH53950.1	77.612	67	8.10E−25
*C. salicis*	KXH52034.1	77.612	67	8.27E−25
*C. fiorinae*	EXF73863.1	77.612	67	8.82E−25
*C. orchidophilium*	XP_022472246.1	76.119	67	7.84E−24
*C. fructicola*	ELA29687.1	77.273	66	3.65E−23
*C. orbiculare*	TDZ15371.1	80.303	66	2.44E−20
*C. trifolii*	TDZ54558.1	78.788	66	7.92E−20
*C. sidae*	TEA20600.1	78.788	66	7.92E−20
*C. incanum*	KZL82629.1	79.412	68	6.78E−19
*C. incanum*	OHW94975.1	79.412	68	7.11E−19
*C. spinosum*	TDZ13928.1	74.242	66	2.22E−18
*C. gloeosporioides*	3DCN_A	93.939	33	3.07E−12

#### 3.2.4 Conservation of cutinase based on 200 fungal amino acid sequences

**Figure 6:**
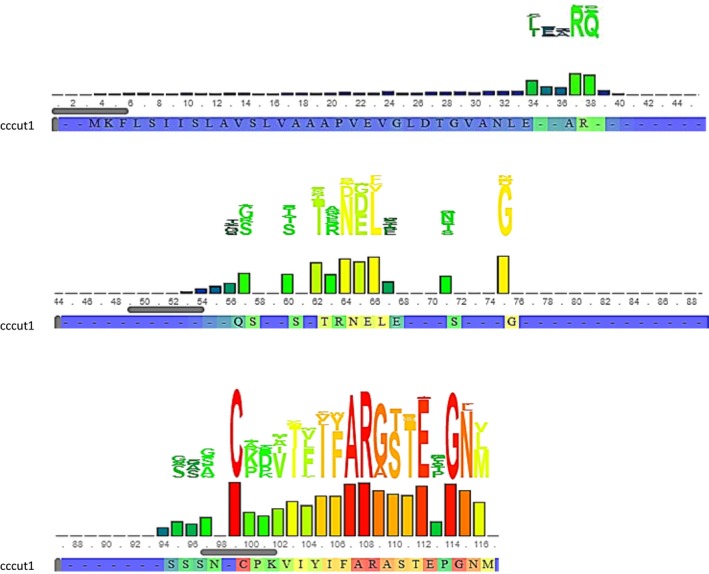
Alignment of 66‐amino acid cutinase domain of 200 different fungal species showing sequence diversity “hot spots” in CATHDB. Columns in the alignment are colored where the highly conserved residues are shown in red through to positions that are not conserved at all, shown in blue

An alignment of 200 truncated amino acid sequences of different fungal species with an identified cutinase domain in CATH‐Gene3D protein data bank revealed specific regions in the sequence that may be indicative of “diversity hot spots” (Figure 6). This is in comparison to the conservation plot of aligned amino acid sequences of *C. truncatum* isolates from Trinidad and two reference *C. truncatum* sequences mined from GenBank which illustrated 100% conservation. The functionally important residues in this protein superfamily are expected to be highly conserved. CATH indicated that the name of this superfamily (3.40.50.1820), previously called alpha/beta hydrolase, has been modified, and the official domain name is pending.

#### 3.2.5 Structure of cutinase gene

The structure of the cutinase gene and protein was analyzed across 34 available *Colletotrichum* genomes in the MycoCosm database. *C. truncatum* and *C. brevisporum* were not among the genomes available for reference in the MycoCosm repository. The predicted cutinase gene length for 34 *Colletotrichum* sequences in the MycoCosm database whose cutinase gene length ranged from 967 bp to 1,296 bp which included two exons interrupted by one intron. Among the 34 genomes, the cutinase gene was composed of two exons interrupted by one intron. Exon 1 varied from 343 to 733 bp in length, exon 2 varied from 556 to 786 bp in length, and the intronic sequence varied in length from 32 to 85 bp. (Figure S1). There was, therefore, one splice variant where the intervening intron between the two coding regions is removed.

#### 3.2.6 Protein signatures

There was a highly conserved domain (Figure S2), characteristic of serine hydrolase, consisting of 194 amino acid residues with a Ser, His, Asp catalytic triad, and 5‐element fingerprint (motifs 1 to 5) among all *Colletotrichum* species. As such, the enzyme had three identified active sites, ACT_SITE140, ACT_SITE195, and ACT_SITE208 (https://www.ebi.ac.uk/enzymeportal/search/P10951/enzyme), and whose source data are derived from IntEnz (Integrated relational Enzyme database; Fleischmann et al., 2004) and UniProt. There was also evidence of two disulfide bonds (amino acid positions 49 to 198 and 129 to 191) which play a critical role in holding the catalytic residues in juxta‐position; reduction of the disulfide bridges results in the complete inactivation of the enzyme and, therefore, these bonds are involved in posttranslational modification of the enzyme (Figure S2; UniProt P00590, *CUT1*). Verification of the protein structure and function (molecular function was cutinase activity [GO id 0050525; Interpro id IPR011150] and cellular component was extracellular/secreted [GO id 0005576; Interpro id IPR11150]) was carried out in EnsemblFungi (https://fungi.ensembl.org/index.html; Kersey et al., 2015) by comparing linked cutinase protein sequences in the Gene3D, SPRINTS, SMART, and PROSITE repositories. Each “GO” term was linked to the three major categories “Cellular Component,” “Biological Process,” or “Molecular Function.”

Analysis of the cutinase gene for *Colletotrichum* sp. (*C. gloeosporioides* was selected as a model nucleotide sequence, CGGC5_1134; transcript ELA29687, as there were no *C. truncatum* or *C. brevisporum* model gene sequences in the EnsemblFungi database). Orthologue analysis identified 20 orthologues of the cutinase gene with other fungal species purportedly as a result of a gene duplication event that occurred ~ 10 Million years ago prior to any speciation event and whose root species belongs to Pezizomycotina (majority of Ascomycetes and lichenized fungi) (Genomicus v. 30.01, http://www.genomicus.biologie.ens.fr; Nguyen, Vincens, Roest Crollius, & Louis, 2017; Figure S3).

#### 3.2.7 Organization and synteny of cutinase gene among *Colletotrichum* genomes

**Figure 7:**
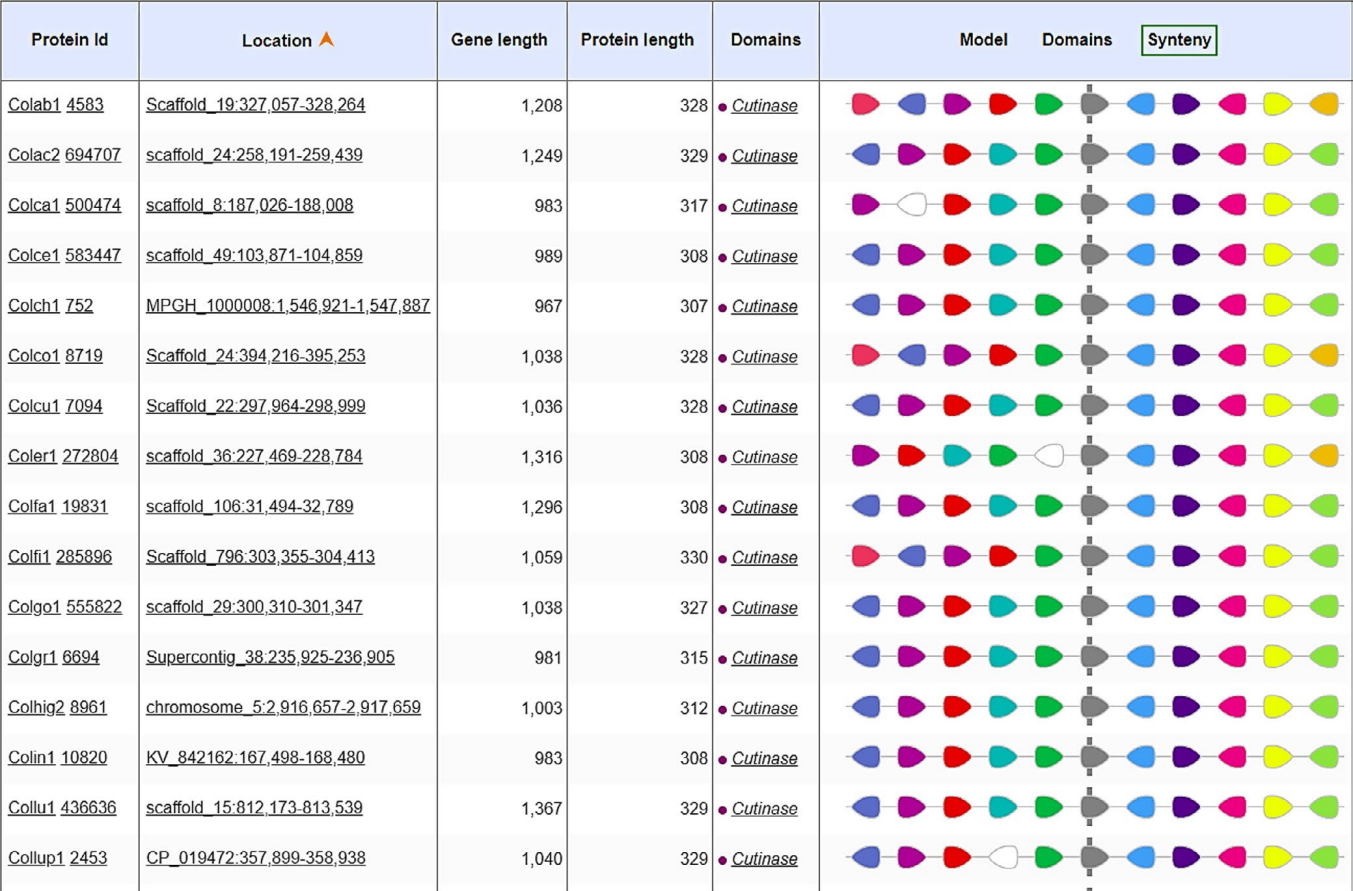
Synteny of cutinase domain and flanking domains in 16 *Colletotrichum* species as identified in MycoCosm

**Figure 8:**
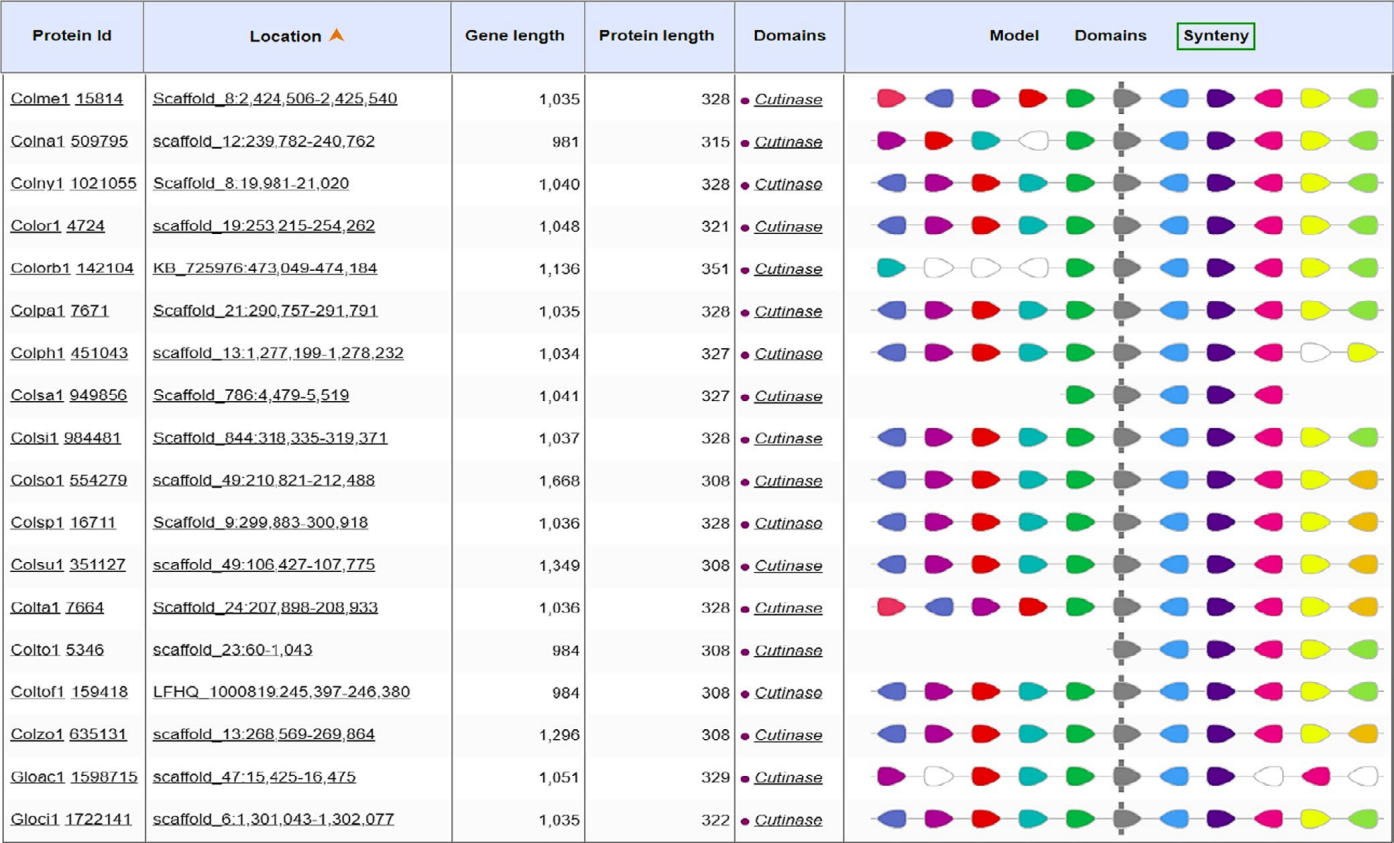
Synteny of cutinase domain and flanking domains in 18 *Colletotrichum* species as identified in MycoCosm

**Figure 9:**
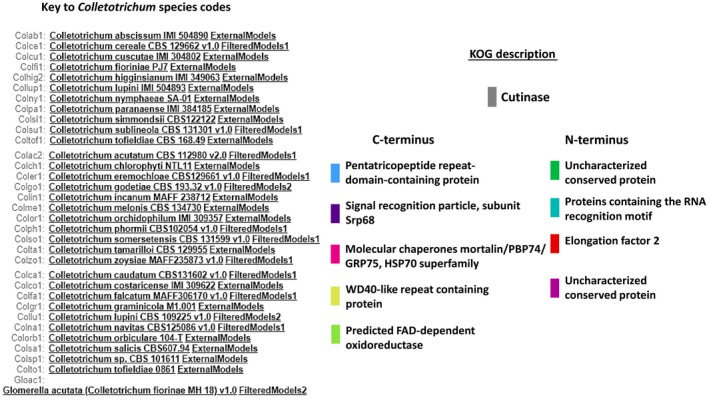
Key for *Colletotrichum* species codes analyzed for synteny of the cutinase domain and the KOG key to these domains as identified in MycoCosm

Synteny analysis in MycoCosm indicated that the cutinase domain was flanked by highly conserved amino acid sequences at both the N‐ and C‐termini in the sequences of 34 *Colletotrichum* species (Figures 7, 8, 9). At the N‐terminus, there was an uncharacterized but highly conserved hypothetical protein (KOG id 2306) whose amino acid sequence identity ranged from 69.3% (GenBank Accession No. EQB49109) to 97.6% (GenBank Accession No. KXH27358). Model testing revealed that 100% of all external track models returned the same uncharacterized but conserved description for this protein domain. SMART (Simple Modular Architecture Research Tool; https://smart.embl.de/; Letunic & Bork, 2017) using hidden Markov models (HMMs) confidently predicted low complexity regions based on Seg analysis (http://mendel.imp.ac.at/METHODS/seg.server.html), in addition to another domain of unknown function, DUF4210. Proteins containing this DUF4210 domain include the animal FAM214A proteins and fission yeast SPAC3H8.04 proteins. An RNA recognition motif (KOG id 2135) in addition to translation elongation factor 2 (aEF2; KOG id 0469) was also identified at this terminus in immediate proximity to the cutinase domain.

At the C‐terminus, a PPR domain (pentatricopeptide repeat) was identified whose function is hypothetical but predicted to involve RNA stabilization and processing as it retains an amino acid sequence‐specific RNA‐binding site (KOG id 4197). Immediate to this PPR domain, is a signal peptide involved in secretion (KOG id 2460). Identification of this signal peptide [VAA‐AP] at position 16 and 17 of a 66‐amino acid sequence, translated from a core coding region of the test isolates’ amino acid sequence, was verified using SignalP 5.0 (http://www.cbs.dtu.dk/services/SignalP/; Armenteros et al., 2019) and Phobius (http://phobius.sbc.su.se/; Madeira et al., 2019) software. The signal peptide prediction is consistent with the database annotation (Figure S4–S6). There was no identified transmembrane domain. Molecular chaperones belonging to the HSP70 family of heat shock proteins and which are involved in posttranslational modification and protein folding (KOG id 0102) were located closer to the C‐terminus. WoLFPSORT (https://www.genscript.com/wolf-psort.html; Horton et al., 2007) was used in protein subcellular localization prediction which confirmed that the enzyme product, deduced from in‐frame amino acid sequence of test isolates, is extracellular or secreted. The identified domains at the both termini were conserved both in their structure and strict arrangement immediate to and relative to the cutinase domain.

## 4 DISCUSSION

This study investigated the diversity, structure, organization, and synteny of a single cutinase gene within *C. truncatum* and *C. brevisporum* isolates from bell pepper fruit in Trinidad and among different *Colletotrichum* species. Evidence of secreted cutinase capability was first tested among the *C. truncatum* and *C. brevisporum* isolates from Trinidad based on bioassays using different lipid substrates.

### 4.1 Lipid substrate bioassays

Bioassays confirmed the ability of *C. truncatum* and *C. brevisporum* to produce and secrete cutinase enzyme in lipid substrate media. Growth characteristics for the identification of secreted lipase activity using Tween‐20, Rhodamine, and tributyrin agar plates were congruent with the results obtained from other studies (Lanka & Latha, 2015; Ramnath, Sithole, & Govinden, 2017; Wadia & Jain, 2017). As both Tween‐20 and Tributyrin can test positive with esterases, the positive fluorescence with Rhodamine B was used as the determining factor for the presence of lipase activity (Lanka & Latha, 2015; Tomulescu et al., 2015). Bornscheuer (2002) explained that lipases can be differentiated from carboxyl esterases based on their substrate spectra: *p*‐nitrophenyl palmitate (*p*NPP) is hydrolyzed by lipases; *p*‐nitrophenyl butyrate (*p*NPB) is hydrolyzed by esterases. However, Chen, Liang, Zhang, and Rodrigues (2006) reported that *C. kahawae* and *C. gloeosporioides* isolates demonstrated cutinase activity on tributyrin media but this activity was significantly reduced with *p*‐nitrophenyl butyrate assays. Conversely, Bonnen and Hammerschmidt (1989) stated that a purified cutinase enzyme, extracted from *C. lagenarium* isolates, was active on both cutin and *p*‐nitrophenylbutyrate as substrates. For this reason, three bioassays using different lipid substrates were used to assess cutinase activity in this study.

### 4.2 Number of cutinase genes in *C. truncatum* compared with other fungal genomes

Only one cutinase gene has been identified in *C. truncatum* (Auyong et al., 2015), and this gene was the target under study. Primers were designed to amplify a portion of this cutinase gene for genetic analysis of *C. truncatum* and *C. brevisporum* isolates infecting bell pepper in Trinidad. Most plant pathogenic fungi possess multiple copies of genes that encode a family of cutinase enzymes: 14 to 17 cutinase genes in *Magnaporthe oryzae*, 13 in *Curvularia lunata*, 13 in *V. dahliae*, 12 in *F. graminearum*, 11 in *Botrytis cinerea*, seven in *Rhizoctonia solani,* and six in *R. cerealis* (Skamnioti, Furlong, & Gurr, 2008; Liu et al., 2016; Gui et al., 2018; Lu, Rong, Massart, & Zhang, 2018). The adaptive advantage of multiple gene copies may include (a) an increase in the amount of gene product generated, (b) copies of the same gene may retain their original functions, but are expressed at different levels and at different times in response to different triggers, (c) one or more copies of the gene can evolve to carry out a new important functional role (neofunctionalization), so that conservative selection acts to preserve multiple versions of the gene (Altenhoff et al., 2019).

### 4.3 Cutinase nucleotide and amino acid sequence variation among only *C. truncatum*

Cluster analysis indicated a low level of nucleotide variation among *C. truncatum* sequences. Wang et al., (2017) also found high homology among cutinase (*CUT1*) gene sequences of *C. gloeosporioides.* The cutinase nucleotide sequences of *C. brevisporum* in this study, were more related to *C. truncatum* cutinase nucleotide sequences than to *C. gloeosporioides*. Cluster patterns coincided with haplotype with moderate to high bootstrap support. Zhu and Freeland (2006) purported that the degenerate nature of the genetic code enables amino acid changes that conserve certain amino acid properties. As such, the 66‐amino acid sequence translated from the partial coding region of the cutinase gene was highly conserved within *C. truncatum* and *C. brevisporum* species. Pathogenesis is actualized through cutinase production and secretion among *C. truncatum* isolates, therefore, evolutionary maintenance of those amino acids in the primary protein structure that are important for folding, structural stability, and are required to form a substrate binding site(s) which guarantees the catalytic ability of the enzyme (Altenhoff et al., 2019; Rudnicki, Mroczek, & Cudek, 2014).

### 4.4 Cutinase sequence diversity

The cutinase gene family in various genera of fungal genomes demonstrates a high degree of coding‐sequence variation reflective of the diverse of roles attributed to cutinases and which perhaps, allowed adaptation to diverse ecological niches over time (Deng, Carbone, & Dean, 2007; Skamnioti et al., 2008). However, the relative degree of nucleotide and amino acid sequence diversity in addition to the role of these genes and their gene products in pathogenesis can vary (Liu et al., 2016; Skamnioti & Gurr, 2007; Sweigard, Chumley, & Valent, 1992). For example, in *M. oryzae*, *CUT1* gene is essential for pathogenicity and *CUT2* gene is required for cuticle sensing and formation of infection structures (Skamnioti et al., 2008). It is also reasonable to associate comparatively high cutinase gene family diversity in the genomes of *M. oryzae*, *B. cinerea,* and *F. graminearum* because of their alternate saprophytic/pathogenic lifestyle; wide host range and concomitant exposure to cutin from a number of host plant species and their subsequent acquired ability to sense and degrade cuticle in different plant species and organ types. In this study, diversity hot spots were identified in the cutinase amino acid sequence when 200 fungal species were compared and this could be explained by divergent evolution allowing different functions of cutinase enzyme in plant pathogenic fungi. Gui et al. (2017) conducted functional analysis of 13 cutinase genes (VdCUTs) and found significant sequence divergence in cutinase family members in the genome of *Verticillium dahliae* Vd991.

### 4.5 Cutinase gene structure within *C. truncatum* and among *Colletotrichum* sp

Within *C. truncatum* sequences, the cutinase gene structure as cutinase 1 (*CUT1*) was maintained as two exons with a single intervening intron which is spliced out during posttranscriptional modification resulting in a single splice variant based on comparative clustering. The relative positions of the two exons and the intervening intron varied within base pairs among *Colletotrichum* species and this is in keeping with the findings of Liu et al. (2016).

### 4.6 Orthologue detection in 200 fungal genomes and implications of gene duplications

Twenty cutinase orthologues detected among different fungal species and whose common ancestor is Pezizomycotina were identified in this study. Using the CATH‐Gene3D hierarchical classification approach which grouped cutinase protein domains according to sequence, structure, and functional diversity, confident predictions of the likely evolutionary relatives indicated that a hypothesized gene duplication event is one mechanism by which the cutinase gene in *C. truncatum* evolved. Gene duplication is an evolutionary tool by which new genes and genetic novelty can be generated in eukaryotes which in turn, would serve to increase the plasticity of a genome or species as an adaptive advantage to changing environments. Several factors impact upon whether a duplicated copy of this gene is fixed in the lineage of given pathogen population through positive selection forces. Primarily, the copy must serve some selective advantage to the pathogen, for example, two copies of one gene may increase the rate of expression and production of gene products or the duplicated copy may have evolved through neofunctionalization and is now adjunct to the function of the original gene (Magadum, Banerjee, Murugan, Gangapur, & Ravikesavan, 2013; Savory, Leonard, & Richards, 2015). Among test isolates in this study, the aligned cutinase nucleotide sequences, generated from the cccut‐F/R primer pair, demonstrated signatures of significant positive selection according to Fu and Li's D* statistic which indicates the importance of this gene's functions during host pathogen interaction (Tiffin & Moeller, 2006).

It is suggested that the hypothesized lineage of the cutinase gene emerged after its transference between distantly related plant‐degrading microbes through lateral gene transfer events (LGTs) (Belbahri et al., 2008). Genes that evolved from what is considered to be an “ancient” duplication event (i.e., duplication before speciation as in the case of Pzezizomycotina and subsequent speciation events that occurred after a single duplication event) may have diverged to an extent that created new functions (Altenhoff et al., 2019). Gene function annotation of 13 *Colletotrichum* species with 11 other fungal species indicated that the evolutionary features involved in pathogenesis of *Colletotrichum* fungi existed at multiple taxonomic levels, some of which were facilitated by fungus‐to‐fungus lateral transfer which suggests that the success of *Colletotrichum* fungi as plant pathogens are due in part to shared, as well as lineage‐specific virulence factors (Liang et al., 2018). These findings provide insight into the acquisition of virulence factors in these important plant pathogens (Belbahari et al., 2008).

### 4.7 Synteny among 34 *Colletotrichum* genomes

It is useful to investigate synteny, in the context of genome structure evolution, because it demonstrates the comparative framework by which conservation of protein‐coding genes and gene order are enabled between genomes of different species. This type of analysis is carried out under the presumption that genome assembly of good contiguation is used in the analysis (Liu, Hunt, & Tsai, 2018). The findings revealed that the cutinase domain was retained both in structure and arrangement among *Colletotrichum* species. The order of aligned genomic blocks between species and the arrangement of flanking protein domains were also conserved and shared for those domains immediately located at the N‐ and C‐terminus of the cutinase domain. No synteny is suggestive of genomic rearrangements during speciation in order to colonize different niches (Bhadauria, Vijayan, Wei, & Banniza, 2017). Among the conserved domains that flanked the cutinase block were signal peptide and chaperone proteins. One explanation for this finding is the production of cutinase is enhanced by signal peptide optimization and chaperone expression (Yao, Su, Li, & Wu, 2019). It is important that the function of the highly conserved region located at the immediate N‐terminus of the cutinase domain found in this study, be characterized because its high interspecific conservation in arrangement strongly suggests a contributory role in cutinase expression and function among *Colletotrichum* species. Further, these conserved genomic blocks can be alternative targets for cutinase function disruption and possibly disease control.

In this study, it was found that positive selection of low diversity cutinase gene sequence, preservation of amino acid sequence with conservation of protein function and synteny have been identified as evolutionary mechanisms that maintain the cutinase gene in the genomes of *Colletotrichum* species. This information is important to developing strategies to enhance plant defense and decrease the pathogenic potential of fungal phytopathogens by targeting and disrupting the cutinase genomic block identified in this study for *Colletotrichum* species (Brunner et al., 2013). This may be particularly important for the management of *C. truncatum* diseases as there is only one cutinase gene in *C. truncatum* available for targeted gene disruption.

## CONFLICT OF INTEREST

The authors declare no conflict of interest.

## AUTHOR CONTRIBUTIONS

RTV: Conducted the experiments, analyzed the data, and wrote the manuscript; SNR: Conceptualized the experiments, analyzed the data, and wrote the manuscript.

## Data Availability

DNA sequences: Genbank accessions numbers: MG822830‐2, KJ780718, KR029613, MG827234‐5, MG839690‐1, MG870320‐1, MN473062, and MN473063.

## APPENDIX 1

**Table A1:** Frequency count with respect to the location of lesions caused by *Colletotrichum truncatum* on green bell pepper fruit

Location on the Fruit	Count (yes)	Count (no)
Shoulder	29	23
Face	26	26
Bottom	24	28

**Table A2:** Summary of statistics for colony diameter (mm) for each *Colletotrichum* species on Tween‐20

	*Colletotrichum truncatum*	*Colletotrichum brevisporum*
Mean	19.5379	11.5925
*SD*	6.58016	1.85959
SE Mean	1.34316	0.75917
Minimum	13.22	9.21
Maximum	31.84	13.62

**Table A3:** Analysis of variance (ANOVA) of colony diameter (mm) values for *Colletotrichum truncatum and C. brevisporum* on Tween‐20

	SS	DF	MS	F	*P*
Between	303.022	1	303.022	8.375	0.007
Within	1,013.147	28	36.184		
Total	1,316.169	29			

Note

DF, Degrees of freedom; F, *F* statistic; MS, Mean square; *P*‐Significance level at *p* ≤ .05; SS, Sum of squares.

**Table A4:** Summary of statistics for colony diameter (mm) for each *Colletotrichum* species on tributyrin

	*C. truncatum*	*C. brevisporum*
Mean	6.1815	0.567
*SD*	6.05387	0.12695
SE Mean	1.23574	0.05183
Minimum	0	0
Maximum	15.56	0.32

**Table A5:** Summary of statistics for halo size (mm) for each *Colletotrichum* species on tributyrin

	*C. truncatum*	*C. brevisporum*
Mean	8.7121	8.8267
*SD*	3.0399	2.3646
*SE* mean	0.6205	0.94977
Minimum	3.42	6.32
Maximum	12.74	12.28

**Table A6:** Analysis of variance (ANOVA) of colony diameter (mm) values for *C. truncatum and C. brevisporum* on tributyrin

	SS	DF	MS	F	*P*
BETWEEN	180.063	1	180.063	5.981	0.021
WITHIN	843.017	28	30.108		
TOTAL	1,023.080	29			

Abbreviations: DF, Degrees of freedom; F, *F* statistic; MS, Mean square; *P*‐Significance level at *p* ≤ .05; SS, Sum of squares.

**Table A7:** Analysis of variance (ANOVA) of halo size (mm) values for *C. truncatum and C. brevisporum*

	SS	DF	MS	F	*P*
BETWEEN	0.063	1	0.063	0.007	0.932
WITHIN	239.430	28	8.551		
TOTAL	239.493	29			

Abbreviations: DF, Degrees of freedom; F, *F* statistic; MS, Mean square; *P*‐Significance level at *p* ≤ .05; SS, Sum of squares.
