# Supplementary material for: Diversity, structure, and synteny of the cutinase gene of Colletotrichum species
Source: Ecol Evol. 2020 Jan 21;10(3):1425–43. doi: 10.1002/ece3.5998 (PMC7029052; doi:10.1002/ece3.5998)
Supplement: Supplementary file 1 [file ECE3-10-1425-s001.docx]

**Supplementary Figures**


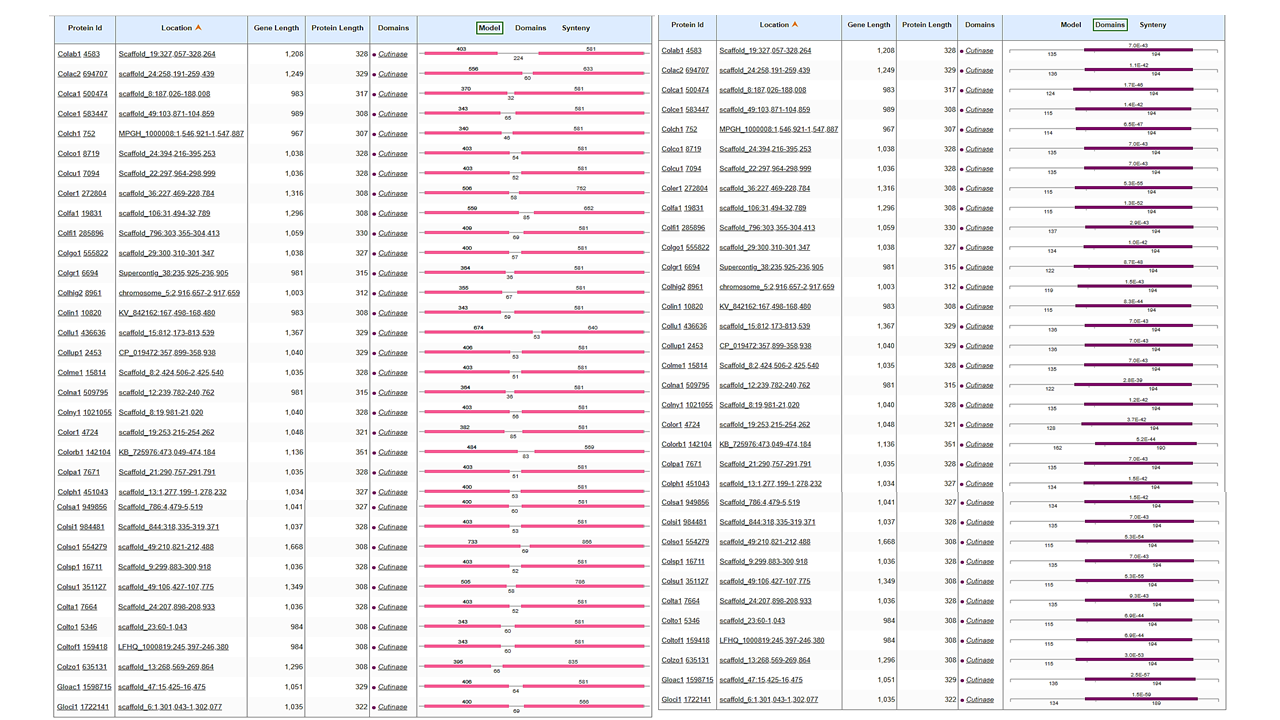


Figure S1 – Cutinase gene structure, domain and location in 34 *Colletotrichum* species as identified in MycoCosm


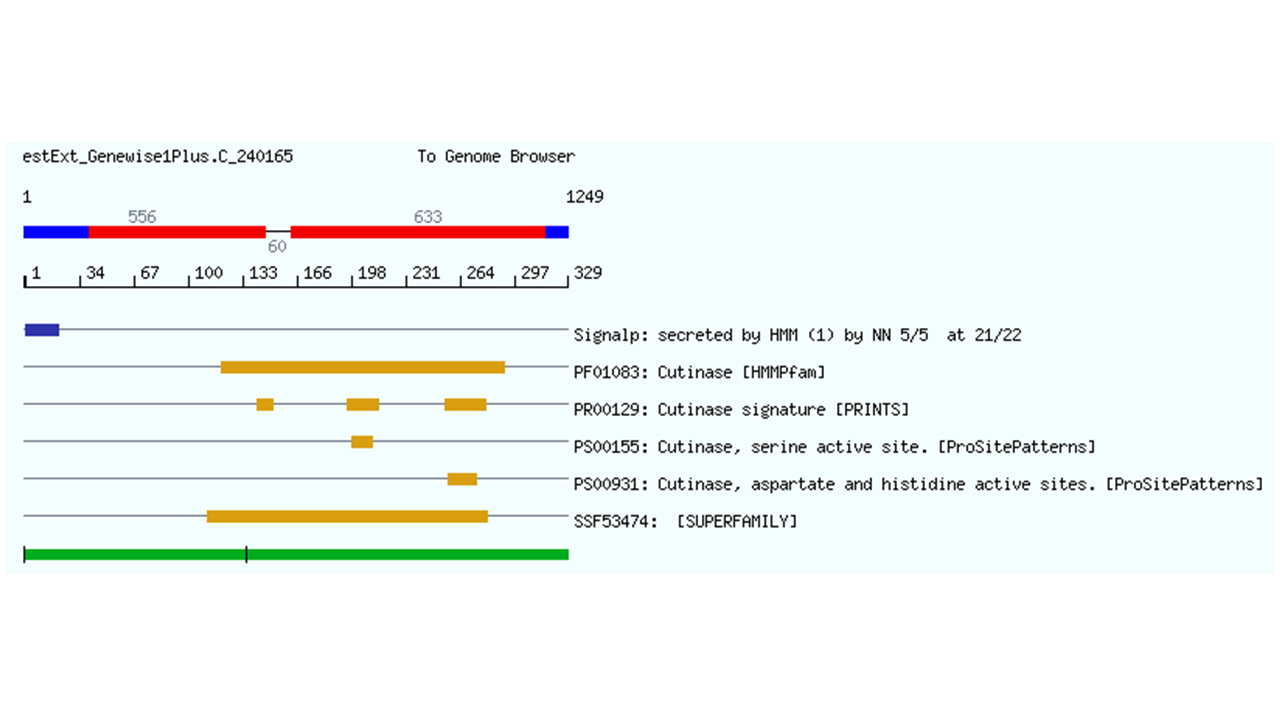


Figure S2 – Map of protein signatures in cutinase gene common to 34 *Colletotrichum* species


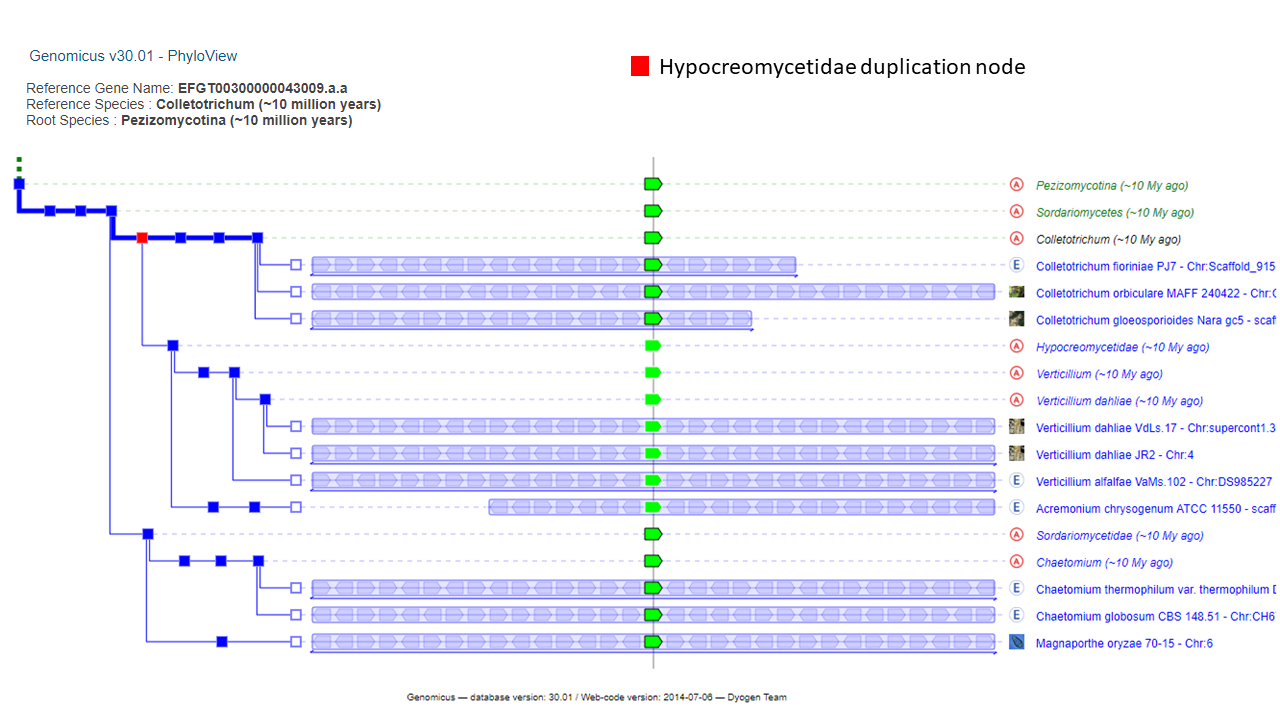


Figure S3– Gene duplication event and cutinase orthologues identified in *Colletotrichum* species in Genomicus


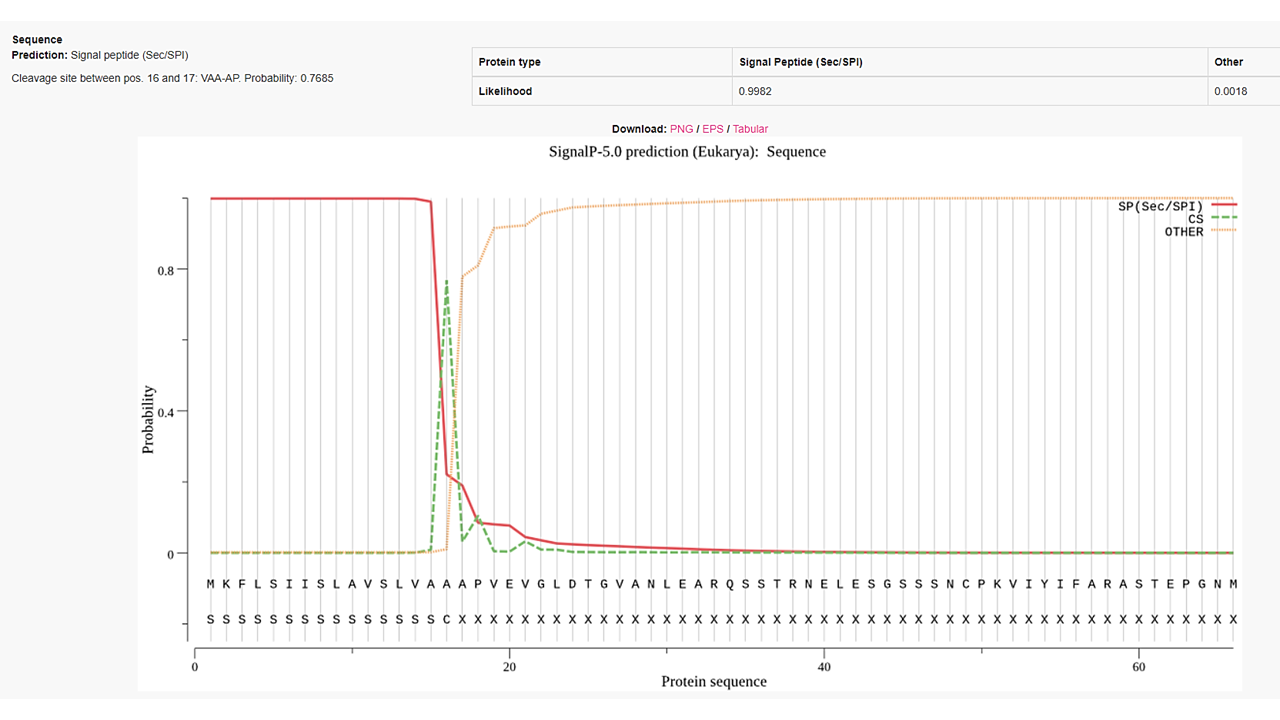


Figure S4 – Signal peptide detection in SignalP


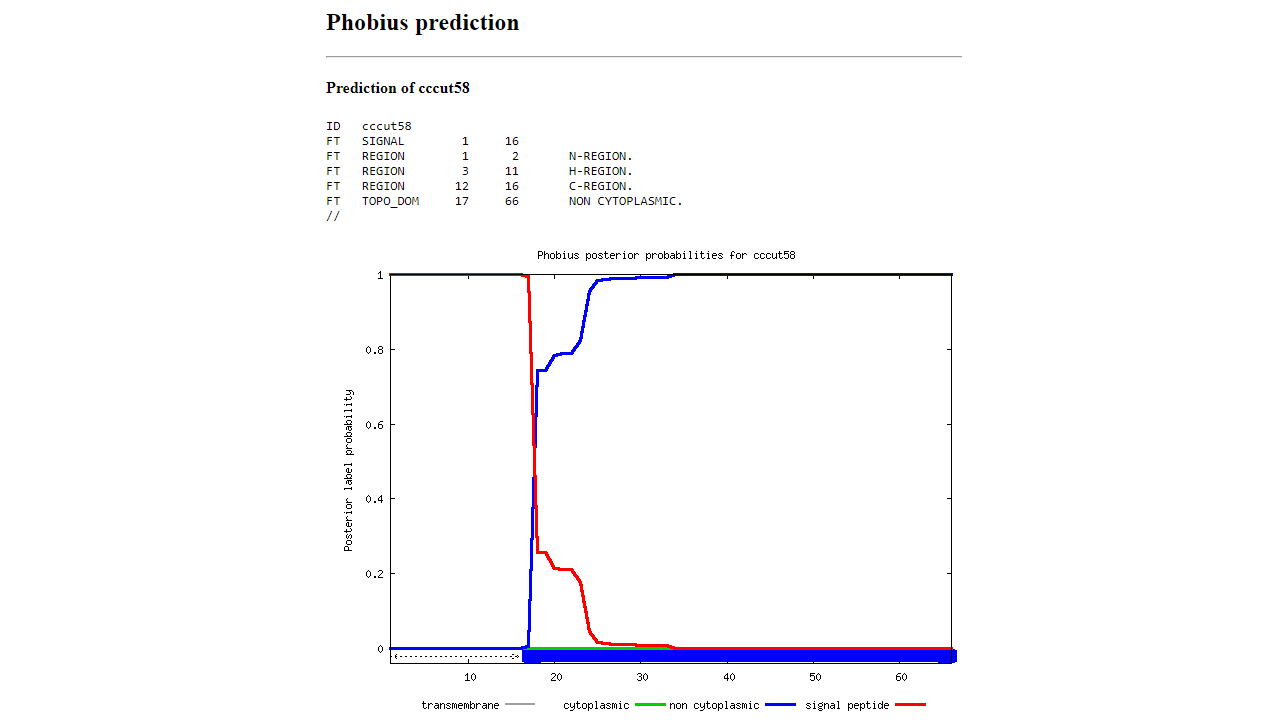


Figure S5 – Signal peptide detection in Phobius


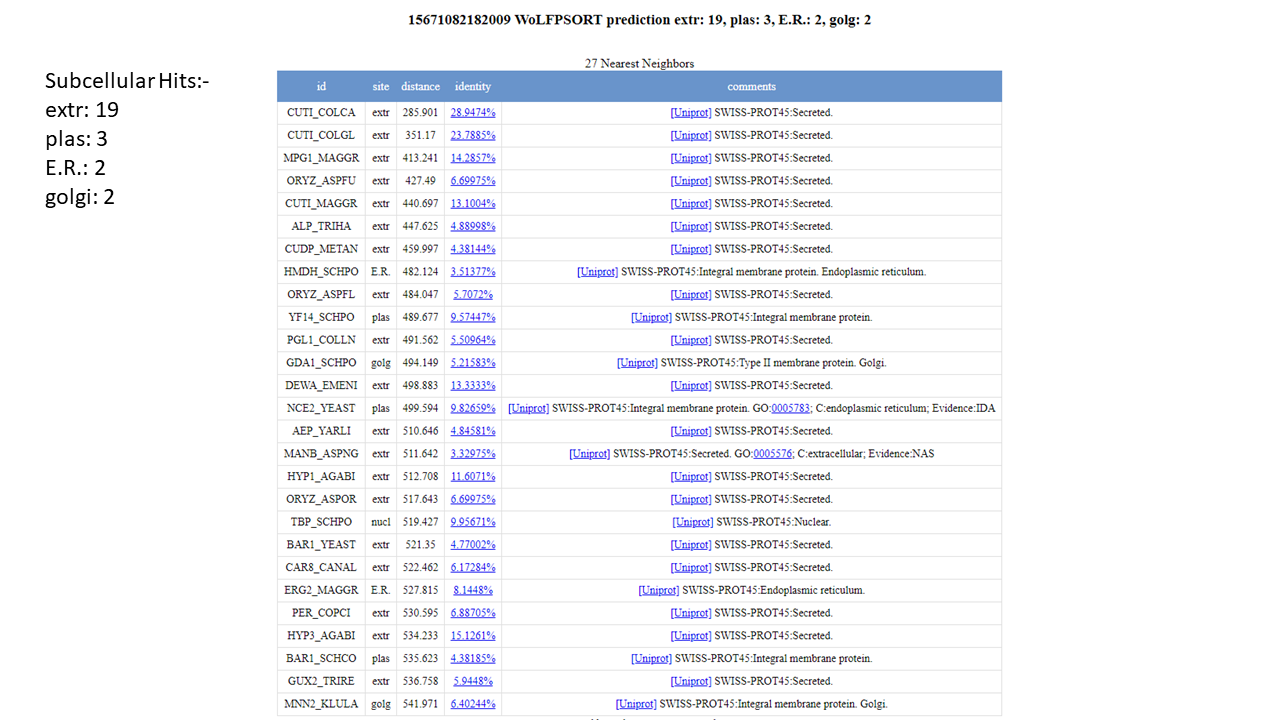


Figure S6 – Signal peptide detection in WoLFPSORT
